# Mitogenomic sequencing of the Brazilian Mastiff and Brazilian Terrier suggests a complex scenario of breed formation for two established Brazilian dog breeds

**DOI:** 10.1590/1678-4685-GMB-2025-0149

**Published:** 2026-04-17

**Authors:** Thaís Fontenelle, Pedro Côrtes, Carolina Furtado, Francisco Prosdocimi

**Affiliations:** 1 Universidade Federal do Rio de Janeiro, Instituto de Bioquímica Médica Leopoldo de Meis, Laboratório de Genômica e Biodiversidade, Rio de Janeiro, RJ, Brazil.; 2 Instituto Nacional de Câncer, Departamento de Genética e Virologia Tumoral, Rio de Janeiro, RJ, Brazil.

**Keywords:** Brazilian Terrier, Brazilian Mastiff, canine mitogenomics, dog domestication, phylogenomics

## Abstract

Brazil has two dog breeds recognized by the Fédération Cynologique Internationale: the Brazilian Terrier and the Brazilian Mastiff. The Brazilian Terrier is believed to descend from Jack Russell Terriers crossed with local strays and possibly Pinschers, while the Brazilian Mastiff is thought to have originated from crosses involving English Mastiffs, Bloodhounds, and English Bulldogs. Here, we partially sequenced the genomes of one pedigree-certified individual from each breed using Illumina HiSeq. We assembled and annotated their complete mitochondrial genomes and performed comparative phylogenomic analyses. The Brazilian Terrier showed the highest mitogenomic similarity to the Australian Shepherd, Miniature Dachshund, Rottweiler, Cairn Terrier, and Shetland Sheepdog. For the Brazilian Mastiff, the closest matches included the Schipperke, Walker Hound, Tibetan Spaniel, Bolognese, and Great Pyrenees. Analysis of the mitochondrial D-loop region confirmed these results with minor variations. Additionally, we analyzed a partial sequence of the *MLPH* gene in the Brazilian Terrier to document genetic variants associated with coat color dilution. Altogether, our findings indicate that the genetic origins of both Brazilian breeds are more complex than traditionally assumed. Future studies with broader sampling and nuclear sequencing will be essential to deepen our understanding of their ancestry and evolutionary relationships.

## Introduction

Dogs (*Canis lupus familiaris*) were likely the first animals domesticated by humans (Clutton-Brock, 1995), with estimates placing their domestication between 15,000 and 135,000 years ago (Vilà *et al.*, 1997; Savolainen *et al.,* 2002). Both genomic and paleontological evidence trace their origins to the Eurasian grey wolf (*Canis lupus lupus*). These two taxa belong to the Canidae family, which currently comprises 38 species, all of them carnivorous. While some canids hunt in coordinated social groups, others are solitary predators. They communicate using facial expressions, body and tail postures, and a wide range of vocalizations, including barking and howling (Clutton-Brock, 1995). Over millennia, humans have shaped dog populations through artificial selection, generating over 1,000 breeds with distinct and often isolated gene pools, resulting in remarkable phenotypic diversity in size, color, and behavioral traits. Dogs have been selectively bred to perform a wide variety of roles, such as hunting, herding, guarding, retrieving, pulling, detecting explosives, drugs, firearms, or diseases like cancer and diabetes, assisting individuals with disabilities, rescuing victims, or simply offering companionship. This process of selective breeding represents an early and intuitive form of biotechnology (Prosdocimi, 2025).

Every year, new dog breeds are created for different purposes, while others go extinct for a variety of reasons. A breed is typically defined by a combination of phenotypic traits, genetic lineage, and a set of breed standards established by recognized breeding organizations. Most breeds can be recognized by their appearance and have strict standards for size, shape, color and temperament. Others are better defined by their instincts or selected function more than their appearance, such as the Dogue Brasileiro and the English Shepherd.

Kennel Clubs are organizations that are responsible for the registration of breeds, keeping official breeding records in a registry and emission of pedigree documents. Most countries have their own Kennel Clubs, but a few are international. The “Federation Cynologique Internationale” (FCI) is perhaps the most reputable international canine organization, with members from 94 countries and 344 breeds recognized. In Brazil, the main entity responsible for breed registration is the Brazilian Confederation of Cynophilia (CBKC), which currently recognizes eight Brazilian dog breeds, two of which are also recognized by the FCI: (i) the Brazilian Mastiff (B. Mastiff) and (ii) the Brazilian Terrier (B. Terrier). 

Both breeds have existed for over a hundred years, but unfortunately the history of their development has never been thoroughly documented. So far, we have relied on a few historical documents, memories and phenotypic similarities to try to determine which breeds were used in their creation.

## The Brazilian Terrier

For the origin of B. Terrier, it has been proposed that the old Jack Russell Terrier (similar to the now called Parson Jack Russell) was crossed with small stray dogs, and possibly with Pinschers or Chihuahuas (Morris, 2001). According to this theory, small terrier dogs were brought to Brazil in European ships. At that time, it was a tradition for young Brazilian upper-class students to study in Europe, and upon return they would often bring back small dogs. Most of the theories determine the dogs that gave origin to the B. Terrier were brought from England (as the Jack Russell), though some believe they were actually brought from Spain, as the Spanish dog breeds Ratonero Bodeguero Andaluz or Ratonero Valenciano. 

The B. Terrier is part of a group of dogs that were bred to hunt and kill small animals that make their housing below ground. Some classifications call these dogs “the terrier group”, which can be misleading, as some Terriers were not bred for this function: the Bull Terrier for example, was originally bred to fight, and the Japanese Terrier was created as a companion dog (Morris, 2001). Additionally, some dogs that do not have the word “Terrier” in their name, such as Dachshunds, some Pinschers and Schnauzers, were also created for hunting vermin and would fit in this group.

These dogs were created to locate quarry by scent or sight, chase them down and kill them. Ideally, they should not eat, mutilate, or even return the prey to the owner, but just drop it. This is one of the most diverse groups of dog breeds, and there are dogs specialized in many types of quarry, adapted to hunt in different terrains and conditions. They are very effective at exterminating vermin, and one of the most effective ways to do so. Some dogs in this group can be very vocal, which makes them great alert dogs. In particular, the B. Terriers are notable at hunting mice, and effective watchdogs. They hunt in packs and, besides mice, also hunt armadillos, quail and essentially many sorts of local small mammals. 

In 1981, the Clube do Fox Paulistinha (as the B. Terrier is known in some regions of Brazil) was formed, and due to the efforts of its members, the breed was recognized by the FCI in 1995 on a provisional basis, and officially in 2007. With international recognition, dogs were exported to different places in Europe, North and South America. Physically, the B. Terrier’s body is medium sized and slender, its head is shaped in a triangular form and the stop is well pronounced. Some individuals are born with no tail (bobtail), otherwise the tail was traditionally docked (a controversial practice that is currently banned in several countries). The fur is short and soft, and the official standard by FCI recognizes the following colors: black and white, blue and white, brown and white, and isabella and white, all of which must be accompanied by tan marks above the eyes, on both sides of the muzzle, the inner part of the ear and in its borders. The blue and isabella colors are associated with eumelanin dilution related gene, known as the Melanophilin (*MLPH*) gene. If the individual carries two copies of the recessive allele, it is born with the diluted color; blue is the result of diluted black; while isabella is the denomination of the color resulting from diluted brown eumelanin. 

The individual used in this study, Grey, was born with white, tan and blue fur, likely resulting from carrying two recessive alleles for the Melanophilin gene. His pedigree records show he was also born with brachyury (a condition also known as bobtail) a naturally occurring dominant mutation in the *T-box* gene (C189G), which causes a change from isoleucine to methionine at amino acid 63 of the encoded protein (Haworth *et al.*, 2001) that results in a shortened tail. In dogs, no homozygous individuals carrying the C189G mutation were found, suggesting an embryonic lethal condition for the homozygous phenotype (Haworth *et al.*, 2001; Indrebø *et al.*, 2008). In rats, mutations in the *T* gene lead to *in utero* death and developmental anomalies in mesodermal tissues (including the spine and the tail), suggesting an essential role of the *T* gene in mammalian development (Wilson *et al.*, 1995). In most breeds, brachyury is considered a fault, while in a few others it is described in the breed standard, as the Pembroke Welsh Corgi (FCI, 2010) and the Brittany Spaniel (FCI, 2003). Therefore, the C189G mutation is not very common in dogs, with only a few breeds known to carry this mutation, including the Brazilian Terrier, the Jack Russell Terrier and the Australian Shepherd. 

## The Brazilian Mastiff

For the origin of the B. Mastiff, there are a few prevalent theories. The first one is that it originated from the now extinct Cão da Fila Terceira, a Portuguese dog breed native from Ilha da Terceira, an island in the Portuguese coast. In Portuguese, the B. Mastiff is called “Fila Brasileiro”, and the word “Fila” means to hold, as in to hold the prey or cattle. Although this is a valid hypothesis, unfortunately there is no genetic material from the extinct breed available for analysis. The second most widespread hypothesis is that the Fila originated from crossings of the Bloodhound, the English Mastiff and the English Bulldog. This theory is divided in two branches: one that accepts there were outcrossings with stray dogs; and another that thinks only the three previously mentioned breeds were used in the Brazilian breed’s development. The third and last hypothesis is that the B. Mastiff was created from the Engelsen Doggen, a molosser type of dog native from England. The Engelsen Doggen (which translates to English Dog in Dutch) is not exactly a breed, but a type of large, strong stray dog that used to populate the streets of England before its extinction. 

The Brazilian Mastiff’s original function was quite diverse. It was used to hunt large animals such as leopards, to guard cattle and farms, and to eventually trace and recover fugitive livestock. Unfortunately, given the tragic history of slavery in Brazil, it is also believed that this breed was used to track and capture enslaved individuals who had escaped through the forests. The breed is known for its exceptional sense of smell-a trait likely inherited from the Bloodhound, one of the most widely used breeds for tracking and rescuing lost individuals, due to its remarkable olfactory capabilities. 

Known to be extremely loyal, affectionate and protective with its family, the Brazilian Mastiff was bred to be wary and suspicious of strangers. To this day, the breed is mainly used in protection of properties, a function it excels at. It is a calm and relaxed dog that at the same time is fearless and eager to protect the family or cattle when necessary. 

Physically, it is a large sized and muscular dog, able to easily surrender a man or a bovine. The ears are large and dropped, and a black mask is often present in the muzzle, although not mandatory. The accepted colors for the breed are brindle, fawn and black, while the coat should be short and smooth, according to the official breed standard by the FCI (FCI, 2016). Among breeders, there is a consensus that both the English Mastiff and the Bloodhound were used in the development of the breed, although no official historical records were kept. 

## Canine genome studies

The canine nuclear genome is organized in 38 pairs of autosomes and a pair of sexual chromosomes (XX or XY), and the mitochondrial genome is organized in one circular chromosome. The first complete mitochondrial genome of a dog was published in 1998, the 20^th^ species to have its mitochondrial genome sequenced (Kim *et al.*, 1998). The work was performed by South Korean students at the Kyungpook National University, who chose the native Korean breed Sapsaree as the study subject, and it can be found under accession number NC_002008 in the NCBI database. As expected, the authors found that the canine mitochondria are structurally similar to other mammals, composed of 13 open reading frames, 22 tRNA genes, two rRNA genes (12S and 16S) and a regulatory control region (Kim *et al.*, 1998). They also compared this mtDNA with other 19 mitogenomes sequenced at the time and found that the dog is genetically closer to harbor and grey seals the cat, horse or rat. 

The first whole genome sequencing of a dog was published in December 2005 in Nature (Lindblad-Toh *et al.*, 2005). The authors sequenced the complete genome of a Boxer and compared the canine genome with the human and the rat ones. The sequencing was performed using the Whole-Genome-Shotgun (WGS) method, which produced a total of 31,5 million reads. The assembled canine genome was labeled as CanFam1.0 and, in 2011, it has been updated to CanFam3.1 (Hoeppner *et al.,* 2014), which remains the most recent version of the complete canine genome. 

In the years following that pioneer studies, thousands of dogs have had their mtDNA sequenced, purebred or not, including ancient ones. In this study, we used data from different dog breeds (including the Brazilian ones sequenced here) and wild canids. Among the breeds, we included basal ones. Basal breeds are genetically divergent breeds that have basal positions on phylogenetic trees, well-supported by genomic studies (Larson *et al.*, 2012; Parker *et al.*, 2017). In theory, the gene pool of these breeds was kept isolated in the past years, and they are genetically closer to the ancestor grey wolf than other modern dog breeds. 

Parker *et al.* (2017) sequenced partial genomes from 161 dog breeds and analyzed 150,067 informative single nucleotide polymorphisms (SNPs). The resulting cladogram identified 23 well-supported clades, categorized based on geographic origin or behavioral traits. Notably, the Terrier and Mastiff breeds each formed distinct clades. A central question we address in this study is whether the Brazilian breeds conform to these established classifications. Would the Brazilian Terrier cluster with other terriers? Would the Brazilian Mastiff align more closely with scent hounds-given its morphological and behavioral similarities to the Bloodhound-or with the traditional mastiffs? Here, we offer a preliminary investigation into the genetic ancestry of these two emblematic Brazilian dog breeds and present initial insights into their phylogenetic relationships within the broader context of canine diversity.

## Material and Methods

### Blood collection

**Figure 1 -:**
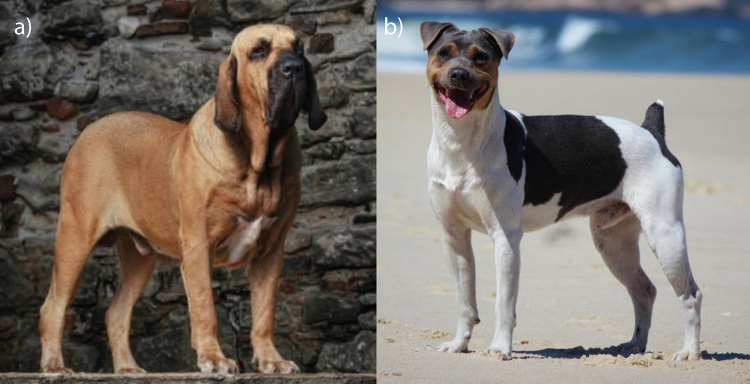
The two dogs sampled for the genome sequencing. (a) The Brazilian mastiff “Boré” (source: Singular Kennel). (b) The Brazilian terrier “Grey” (source: Jardim Imbuí Dog Kennel).

Two individuals (Figure 1) were selected by their accurate representation of the breed standard, as evidenced by their accomplishments in dog shows organized by both national and international kennel clubs. A blood sample was collected from each dog by a licensed veterinarian. The blood was then stored in 4 mL EDTA tubes at a temperature of 4 ℃ for a few days before the DNA extraction. 

### DNA extraction and sequencing

DNA was extracted from the blood samples according to traditional phenol/chloroform nucleic acid extraction protocol (Sambrook and Russell, 2006). The DNA was sequenced through Illumina HiSeq technology, at the National Institute of Cancer (INCA). The two samples were sequenced by an Illumina HiSeq 2500 sequencer.

### Assembly of the Melanophilin gene

The Melanophilin gene was assembled through the MIRA software (Chevreux *et al.,* 1999). The reference contig for the *MLPH* gene was downloaded from the canine genome on the Uniprot database.

### Mitochondrial genome assembly 

The mitochondrial genomes were assembled through *de novo* sequencing on the MIRA v.4.0.2 software with its default parameters (Chevreux *et al.,* 1999). After the initial assemble, the software MITObim v.1.9 (Hahn *et al.,* 2013) was used to perform successive iterations to cover any possible gaps that could have remained after the MIRA assemble, and to assemble a circularized version of both mitochondrial genomes. The iterations were performed with MITObim default settings.

Version 1.17.08.17 of the Tablet software (Milne *et al.*, 2013) with default parameters was used to check the read coverage and circularization of both mitochondrial genomes. The sequencing depth and genome coverage were plotted using a modified version of the algorithm by Ni *et al.* (2023). The drawing of the mitogenome map was done in Proksee (Grant *et al.*, 2023). Annotation was performed automatically through MITOS Web Server (Bernt *et al.*, 2013), which was then followed by manual curation using the Artemis software (Carver *et al.*, 2012). 

### Mitochondrial DNA haplogroup assignment

Haplogroup assignments were determined using an in-house Python script designed to implement the standard cladistic classification of dog mitochondrial DNA (mtDNA) as defined by Fregel *et al.* (2015). This tool identifies the diagnostic mutations within the mitogenome sequence necessary for accurate placement into clades and subclades.

### Phylogenomics

A dataset of complete mitochondrial genomes from selected dog breeds was downloaded from the NCBI GenBank database, alongside with the complete mitochondrial genome of four wild canids as outgroups. The breeds were selected according to their relationship with the Brazilian ones, based on the hypotheses described before (Table 1 and Table 2). Haplogroup information was based on Fregel *et al.* (2015).

To infer the taxonomic identities, a phylogenetic analysis was performed using a supermatrix approach based in the concatenation of all protein-coding sequences (CDSs). The CDSs were aligned using MAFFT (Katoh and Standley, 2013) with its default parameters and the analysis was then conducted with IQTree (Minh *et al.*, 2020) using the Maximum Likelihood method and the TIM2+F+G4 model, which was selected via ModelFinder (Kalyaanamoorthy *et al.*, 2017). The generated trees were tested using 1000 ultrafast bootstrap replicates and the branches were tested using SH-like aLRT with 1000 replicates. Finally, a phylogram was assembled in FigTree. 

This project and its procedures were approved by the Ethics Committee of the Federal University of Rio de Janeiro (Universidade Federal do Rio de Janeiro), under protocol number 074/18.

**Table 1 -:** Breeds selected for comparison of phylogenetic relationship to the B. Terrier and their reasons.

Description	Species	Breed	Reason	Haplogroup	Accession #	Reference
This study	*Canis lupus familiaris*	Brazilian Terrier	-	?	MH105046	This study
Other breeds	*Canis lupus familiaris*	Jack Russell Terrier	Hypothesis 1	A1d1a1a	AY656738	*Unpublished*
		Dobermann		B1a	EU408269	Webb and Allard *et al.* (2009)
		Chihuahua		A4a1	EU408262	Webb and Allard *et al.* (2009)
		Rottweiler	Used to create the Dobermann	A1a1b	KU290638	Strakova *et al.* (2016)
		Ratonero Bodeguero Andaluz	Hypothesis 2	?	OQ340562	*Unpublished*
		Dachshund	Same function	?	KU290984	Strakova *et al.* (2016)
		Miniature Dachshund		A1a1b	EU408286	Webb and Allard *et al.* (2009)
		German Shepherd	Sheepdogs	C1a1	DQ480489	Bjornerfeldt *et al.* (2006)
		Old English Sheepdog		A1a1a3	AY656742	*Unpublished*
		Shetland Sheepdog		B1a2	DQ480500	Bjornerfeldt *et al.* (2006)
		Border Collie		?	KU290951	Strakova *et al.* (2016)
		Pembroke Welsh Corgi	Sheepdogs + T gene mutation	?	OQ340445	*Unpublished*
		Australian Shepherd		A1a1b2	EU408249	Webb and Allard *et al.* (2009)
		Brittany Spaniel	T gene mutation	A1d1a3	EU408257	Webb and Allard *et al.* (2009)
		Border Terrier	Other Terriers	A1a1a4a1a	JF342813	*Unpublished*
		American Pit Bull Terrier		C1a2	EU408293	Webb and Allard *et al.* (2009)
		Staffordshire Bull Terrier		A1a1	KU290585	Strakova *et al.* (2016)
		Airedale Terrier		A1d1a1a	AY656748	*Unpublished*
		Australian Terrier		B1a6	EU408247	Webb and Allard *et al.* (2009)
		Cairn Terrier		A1a1c	EU408264	Webb and Allard *et al.* (2009)
		Kerry Blue Terrier		B1a2	AY656740	*Unpublished*
		West Highland White Terrier		A1f2a	DQ480497	Bjornerfeldt *et al.* (2006)
		Akita Inu	Basal breeds	A1d1a2a	EU408245	Webb and Allard *et al.* (2009)
		Basenji		?	MW051511	*Unpublished*
		Siberian Husky		A2b2	DQ480499	Bjornerfeldt *et al.* (2006)
Outgroups	*Canis lupus*	Gray Wolf		-	NC_008092	Bjornerfeldt *et al.* (2006)
		Iberian Wolf			NC_060652	Ginja *et al.* (2022)
	*Canis latrans*	Coyote			NC_008093	Bjornerfeldt *et al.* (2006)
	*Vulpes vulpes*	Red Fox			NC_008434	Arnason *et al.* (2006)

**Table 2 -:** Breeds selected for comparison of phylogenetic relationship to the B. Mastiff and their reasons.

Description	Species	Breed	Reason	Haplogroup	Accession #	Reference
This study	*Canis lupus familiaris*	Brazilian Mastiff	-	?	MH105047	This study
Other breeds	*Canis lupus familiaris*	Bloodhound	Hypothesis	?	OQ339520	*Unpublished*
		English Mastiff		A1d1a	EU408274	Webb and Allard *et al.* (2009)
		English Bulldog		?	OQ339556	*Unpublished*
		Neapolitan Mastiff	Other Mastiffs	A1e1	EU408290	Webb and Allard *et al.* (2009)
		Tibetan Mastiff		A2a1a1	HM048871	*Unpublished*
		Boxer		A1d1a1a	EU408253	Webb and Allard *et al.* (2009)
		Dogue de Bordeaux		?	OQ339807	*Unpublished*
		French Bulldog		A1a1a2	JF342905	*Unpublished*
		Great Dane		A1f1a1	EU408276	Webb and Allard *et al.* (2009)
		Rottweiler		A1a1b	KU290638	Strakova *et al.* (2016)
		Beagle	Scent hounds	A1*	AY729880	*Unpublished*
		Basset Hound		B1	OQ339373	*Unpublished*
		Italian Greyhound		A1d1a	EU408280	Webb and Allard *et al.* (2009)
		Swedish Elkhound		C2b1	DQ480501	Bjornerfeldt *et al.* (2006)
		Walker Hound		B1a1a	EU408307	Webb and Allard *et al.* (2009)
		Norwegian Elkhound		D1	EU408288	Webb and Allard *et al.* (2009)
		Akita Inu	Basal breeds	A1d1a2a	EU408245	Webb and Allard *et al.* (2009)
		Basenji		?	MW051511	*Unpublished*
		Siberian Husky		A2b2	DQ480499	Bjornerfeldt *et al.* (2006)
Outgroups	*Canis lupus*	Gray Wolf	-	-	NC_008092	Bjornerfeldt *et al.* (2006)
		Iberian Wolf			NC_060652	Ginja *et al.* (2022)
	*Canis latrans*	Coyote			NC_008093	Bjornerfeldt *et al.* (2006)
	*Vulpes vulpes*	Red Fox			NC_008434	Arnason *et al.* (2006)

## Results

### Mitogenome assembly

**Figure 2 -:**
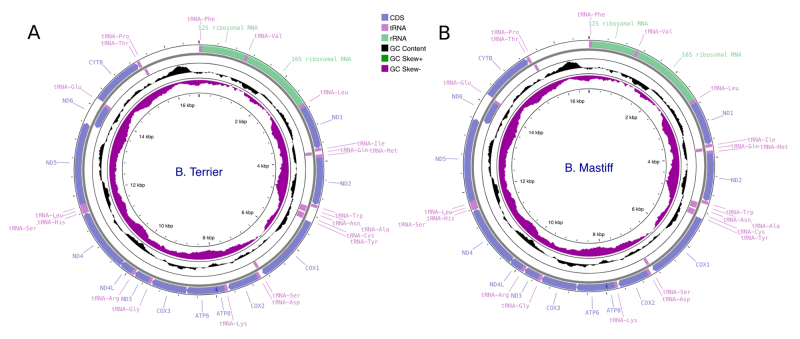
The circular structure of the mitogenome of the two sequenced breeds. Genes on the outside of the outer circle are encoded on the heavy strand, and genes on the inside of the outer circle are encoded on the light stand. Plots of GC skew and content utilized a window size of 500 and reflect GC skew/content on a scale from 0 to 1, with the middle line representing 0.5. Positive and negative skew are indicated by values above and below the midpoint, respectively. a) B. Mastiff; b) B. Terrier.

**Figure 3 -:**
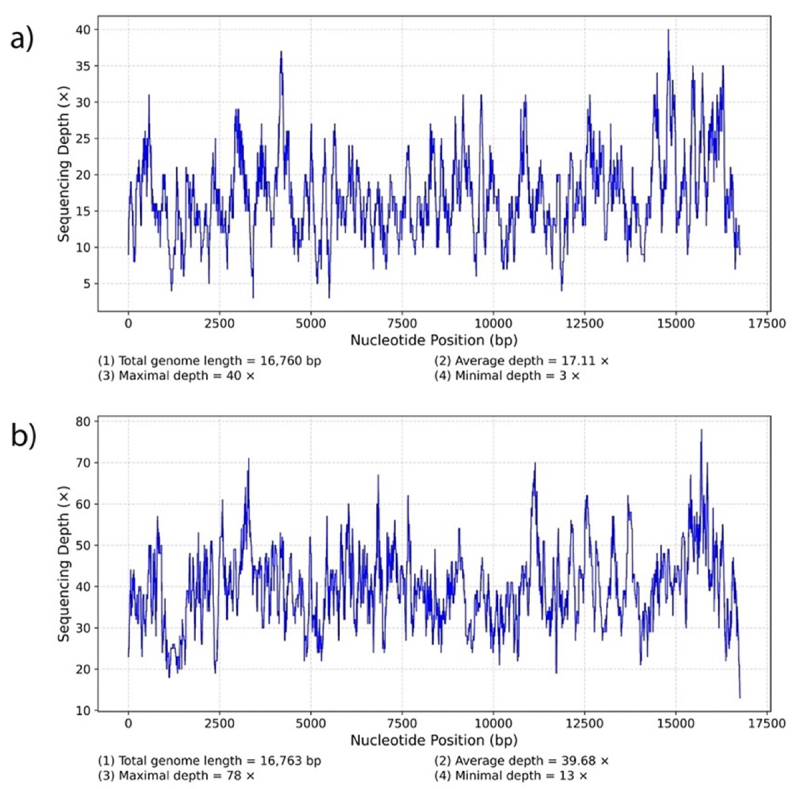
Sequencing depth and genome coverage map for the mitogenomes of the Brazilian Mastiff and Brazilian Terrier sequenced in this study. a) B. Mastiff; b) B. Terrier.

Both mitogenomes were correctly assembled and circularized (Figure 2). For the Brazilian Mastiff, the raw sequencing data resulted in 9,793,966 sequences, of which 1,462 were used in the mitochondrial genome assembly. The average cover was 16.11 and the total length of the mitogenome was 16,732 bp (Figure 3a). For the Brazilian Terrier, we obtained 14,344,120 sequences, of which 4,182 were used in the assembly of the mitogenome (Table 3). The average cover for each position in the mitogenome was 39.68, and the total length comprised 16,730 bp (Figure 3b). The mitogenome of the Brazilian Terrier was 16,730 bp in length and the one from the Brazilian Mastiff was 16,732 bp. Both have sequences representing the codes for the expected two rRNAs, 22 tRNAs and 13 protein coding genes, as well as a control region (D-loop; Supplementary Tables S1 and S2). Using an in-house Python script and the diagnostic mutations defined by Fregel *et al.* (2015), the mitogenome sequences allowed for the precise haplogroup classification of the Brazilian dogs, defining the B. Terrier sample as A1a1b and the B. Mastiff sample as B1a1a (Supplementary Table S3).

**Table 3 -:** Characteristics of the mitochondrial genome sequencing of the Brazilian Terrier and the Brazilian Mastiff.

Feature/ Breed	B. Terrier	B. Mastiff
Number of sequences	14,344,120	9,793,966
Number of bases	1,433,412,000	979,396,600
Number of sequences used in the mitogenome assembly	4,182	1,462
Average genome cover	39.7	16.7
Total size of the mtGenome	16,730	16,732

### Brazilian Terrier

**Figure 4 -:**
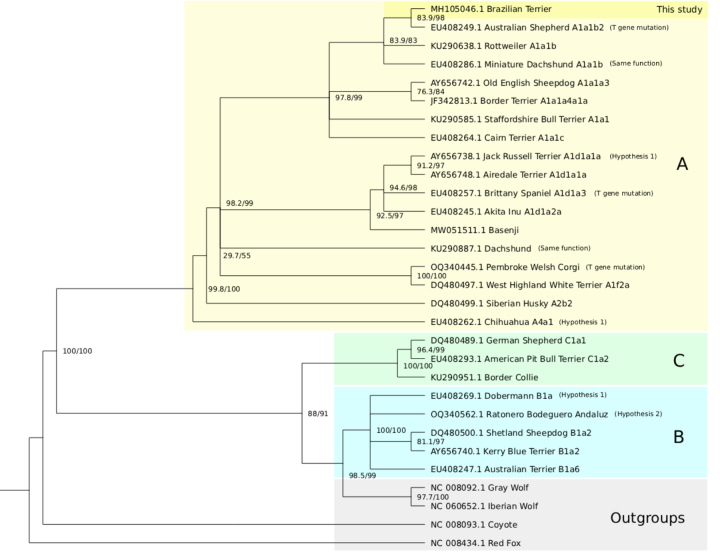
Phylogram of different dog breeds compared to B. Terrier and the haplogroups. In yellow, the A* haplogroup; in green, C*; in blue, B* and in grey, the outgroups. Node numbers are the bootstrap/SH-like aLRT values. Each breed's category and citations are given in Table 1.

The results indicated that B. Terrier did not cluster with other terrier breeds, nor did the terriers themselves form a cohesive group (Figure 4). Instead, the B. Terrier grouped with breeds belonging to haplogroup A1a1b*, showing greatest similarity to the Australian Shepherd - a sheepdog breed often associated with the *T* gene mutation. Other closely related breeds included the Miniature Dachshund and the Rottweiler. Neither of the two main hypotheses regarding the origin of the B. Terrier was fully supported by the data observed here; however, the Jack Russell Terrier, one of the proposed ancestral breeds, also belongs to haplogroup A1*, while the Ratonero Bodeguero Andaluz, another suggested contributor, was assigned to haplogroup B1a*.

According to our analysis, the breeds with the highest percentages of identity with the B. Terrier were: the Australian Shepherd, the Miniature Dachshund and the Rottweiler. We know that few breeds carry the bobtail mutation (when a dog is born without or with only a few vertebrae of the tail), including both the Brazilian Terrier and the Australian Shepherd. We also know that either the Pinscher or the Doberman are good candidates for being used in the creation of the Brazilian Terrier, and the Doberman was created from the Rottweiler. Among the terriers with the lowest percentage of identity with the Brazilian one were the Am. Pit Bull Terrier and the Kerry Blue Terrier. 

The B. Terrier was also placed within the A* haplogroup, alongside the Jack Russell Terrier, suggesting a possible genetic link between the two breeds. In contrast, the Ratonero Bodeguero Andaluz was more distantly related, falling within the B* haplogroup. As outlined by Fregel *et al.* (2015), each haplogroup can be traced through a defined set of mitochondrial mutations. These mutational pathways suggest a shared ancestral lineage between the B. Terrier and the Jack Russell Terrier, with the two likely coalescing before a common ancestor with the Ratonero Bodeguero Andaluz. This could support the hypothesis that the Jack Russell Terrier contributed to the formation of the B. Terrier.

### Brazilian Mastiff

**Figure 5 -:**
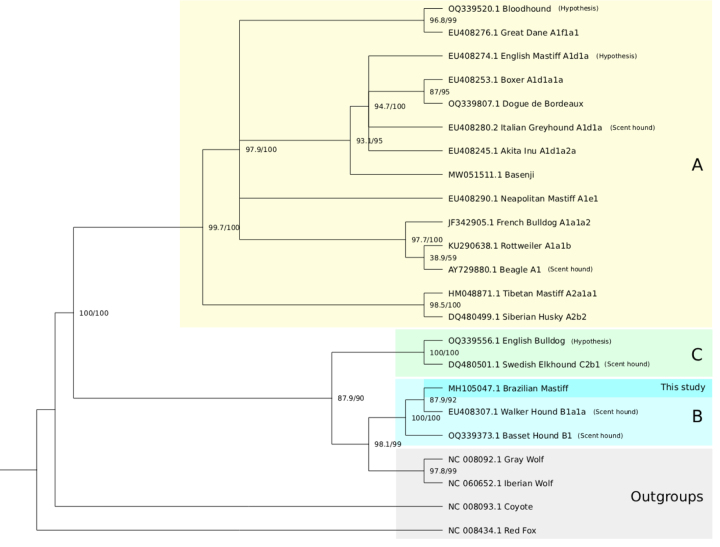
Phylogram of different dog breeds compared to B. Mastiff and the haplogroups. In yellow, the A* haplogroup; in green, C*; in blue, B* and in grey, the outgroups. Node numbers are the bootstrap/SH-like aLRT values. Each breed's category and citations are given in Table 2.

Similarly, the B. Mastiff did not cluster with other mastiff breeds, nor did the mastiffs form a cohesive phylogenetic group (Figure 5). Instead, the B. Mastiff showed the greatest similarity to the Walker Hound and also clustered closely with the Basset Hound - both scent hound breeds that share morphological traits with the B. Mastiff -, which are part of the haplogroup B1*.

For the B. Mastiff, we found that the breeds with highest percentages of identity were: the Walker Hound and the Basset Hound, which are all part of the B* haplogroup. Of these, the Walker Hound was the one expected to have the highest percentage of identity with the B. Mastiff, as it shares both physical and behavioral traits with sighthounds. The haplogroup analysis supports the hypothesis.

### Comparative mitogenomics

Because the analyses are derived from one representative individual of each breed, the results reflect only the maternal genetic lineage and should not be generalized to the entire breed population. When analyzing the phylogenetic trees (Figures 4 and 5), some results were obtained as expected according to Parker *et al.* (2017), such as the Tibetan Mastiff being close to the Siberian Husky. The grouping of haplogroups was exactly as expected based on the work of Fregel *et al.* (2015). However, most phylogenetic relationships were not expected, such as the Australian Terrier being in a different group to the other terriers. Also, many phylogenetic relationships between breeds could not be resolved, as their concatenated sequences were an almost exact match. We attribute these discrepancies to a few factors, such as: (i) the whole mitochondrial genome is not a good tool for observation of intraspecific - or interbreed - relationships, as they are all highly similar; (ii) once we used public data deposited in GenBank, we were not able to control the quality of those sequences; many have shown large gaps and/or significant portions of unidentified bases. In any case, we did filter out the poorly sequenced genomes, but this has certainly had an impact on the results. Also, (iii) we know that the mitochondria come specifically from the maternal lineage; if only either males or females of a certain breed were used in the creation of a second breed, this would affect the results. 

### *MLPH* gene

**Figure 6 -:**
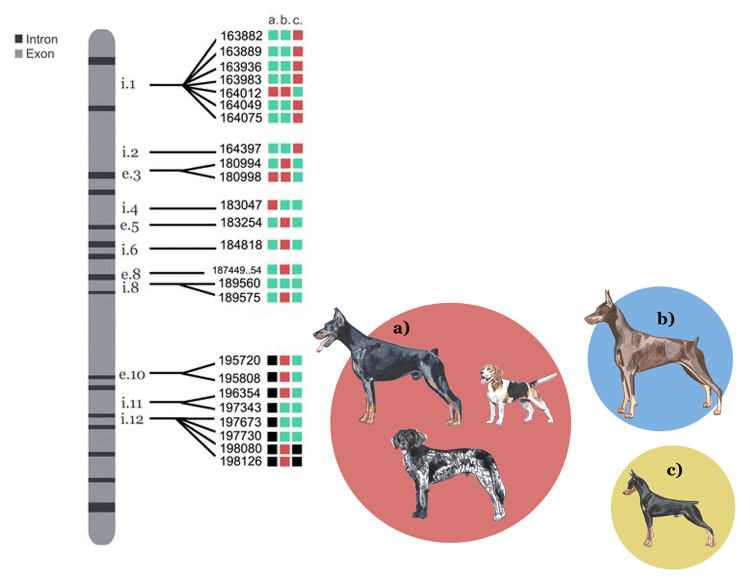
Comparison of SNPs found in the *MLPH* gene of the Brazilian Terrier and other dog breeds. Green = identical, Red = different, Black = gap. Line (a) represents the European-origin Doberman and the Beagle/Large Munsterlander. Line (b) represents the American-origin Doberman, and line (c) represents the German Pinscher.

We also found an incomplete sequence for the *MLPH* gene in the Brazilian Terrier partial genome dataset, and compared it to the version of the European Doberman Pinscher, the Large Munsterlander and the Beagle (which have several of the same SNPs, and therefore are grouped together), the North American Doberman Pinscher, and the German Pinscher (Figure 6). The individual Brazilian Terrier used in this experiment had the diluted color phenotype - resulting in the dilution of the eumelanin present on the coat (known as blue coloration), which is a recessive phenotype - therefore the individual must be recessive homozygous for the *MLPH* gene. With the European Doberman/Large Munsterlander/Beagle, we were able to compare 16 SNPs, 13 of which were identical (81 %). With the North American Doberman, we compared 23 SNPs, 10 of which were identical (43 %). With the German Pinscher, 15 out of 21 SNPs were identical (71 %) (Supplementary Table S4). 

The partial sequence of the *MLPH* gene for the Brazilian Terrier was compared to the sequences of the Beagle/Large Munsterlander/European Doberman Pinscher, the German Pinscher and the North American Doberman Pinscher. We found that the *MLPH* gene in the Brazilian Terrier is most similar to the Beagle/Large Munsterlander/European Doberman Pinscher, followed by the German Pinscher and the North American Doberman Pinscher. These are interesting results, as it is suspected that the Brazilian Terrier originated from a mix of breeds brought from Europe during the Brazilian colonization period, possibly the Doberman Pinscher, as they share some physical traits. The North American Doberman Pinscher was expected to share less similarities in the *MLPH* gene since it originated from European individuals brought to North America that were subsequently bred to produce a subvariation of the breed. These individuals were separated from the European gene pool already some time ago and, therefore, were not expected to have participated in the creation of the Brazilian Terrier. 

## Discussion

In this study, we present the first complete mitochondrial genomes of the Brazilian Terrier and the Brazilian Mastiff, two emblematic dog breeds developed in Brazil and officially recognized by the FCI. Through comparative mitogenomic analyses and preliminary nuclear gene assessment, we explored the genetic affinities of these breeds and evaluated how they relate to established phylogenetic clades defined in previous genomic studies. The small sample size and reliance on mitochondrial genomes used here (N=1 for each breed) restrict the depth of the ancestry inferences. Therefore, our conclusions are intended as preliminary indications of maternal lineage relationships rather than definitive reconstructions of breed origin.

Our results showed that neither the Brazilian Terrier nor the Brazilian Mastiff clustered closely with the breed groups traditionally proposed as their ancestors. The Brazilian Terrier exhibited higher mitogenomic similarity to breeds such as the Australian Shepherd and Miniature Dachshund, rather than to other terriers. Similarly, the Brazilian Mastiff showed closest affinity to scent hound breeds-particularly the Walker Hound and Basset Hound-rather than to other mastiffs. Although the mitochondrial genome may lack sufficient nucleotide diversity and phylogenetic resolution to reliably discriminate between closely related breeds, these unexpected affinities can indicate a more complex and heterogeneous genetic background than previously assumed, likely reflecting historical admixture events and contributions from multiple, undocumented ancestral lineages.

Additionally, we identified a recessive homozygous pattern in the *MLPH* gene associated with eumelanin dilution in the Brazilian Terrier. While a single locus-potentially subject to selection for coat color-is not a reliable marker of breed ancestry on its own, its presence lends modest support to the hypothesis of European genetic contributions to the breed’s lineage-particularly from the Doberman Pinscher and related breeds. 

Altogether, this work offers an initial molecular insight into the genetic ancestry of Brazilian dog breeds, which have long been appreciated for their distinct morphologies and behavioral traits, but remain largely uncharacterized at the genomic level. Although our analyses are based on a limited number of individuals and rely primarily on mitochondrial data, the patterns observed here highlight the importance of integrating molecular tools into breed history reconstruction. Future studies including larger sample sizes of each breed, together with genome-wide nuclear data will be essential to refine and expand our understanding of these unique breeds and their place in the broader context of canine evolution and domestication.

## Supplementary material

The following online material is available for this article:

Table S1 - Characteristics of the mitochondrial genome of a Brazilian Terrier dog.

Table S2 - Characteristics of the mitochondrial genome of a Brazilian Mastiff dog.

Table S3 - Diagnostic mutations supporting mitochondrial haplogroup assignments.

Table S4 - Comparison between SNPs of the Melanophilin gene of a Brazilian Terrier dog and other breeds.
