# Supplementary material for: Mitogenomic sequencing of the Brazilian Mastiff and Brazilian Terrier suggests a complex scenario of breed formation for two established Brazilian dog breeds
Source: Genet Mol Biol. 2026 Apr 17;49(1):e20250149. doi: 10.1590/1678-4685-GMB-2025-0149 (PMC13123249; doi:10.1590/1678-4685-GMB-2025-0149)
Supplement: Table S1 - [file 1415-4757-GMB-49-1-e20250149-s1.pdf]

Genetics and Molecular Biology
https://doi.org/10.1590/1678-4685-GMB-2025-0149

# Supplementary Material to "Mitogenomic sequencing of the Brazilian Mastiff and Brazilian Terrier suggests a complex scenario of breed formation for two established Brazilian dog breeds"

**Table S1 -** Characteristics of the mitochondrial genome of a Brazilian Terrier dog (*Canis lupus familiaris*).

| Gene | Position | | Size | | Codon | | Intergenic Space | |
|---|---|---|---|---|---|---|---|---|
| | Start | End | Nucleotide | Aminoacid | Start | Stop | (bp) | Strand |
| tRNA(Phe) | 1 | 69 | 69 | | | | 0 | + |
| 12S rRNA | 70 | 1023 | 954 | | | | 0 | + |
| tRNA(Val) | 1024 | 1090 | 67 | | | | 0 | + |
| 16S rRNA | 1091 | 2670 | 1580 | | | | 0 | + |
| tRNA(Leu) | 2671 | 2745 | 75 | | | | 2 | + |
| ND1 | 2748 | 3703 | 956 | 318 | ATG | TA- | 0 | + |
| tRNA(Ile) | 3704 | 3772 | 69 | | | | -3 | + |
| tRNA(Gln) | 3770 | 3843 | 74 | | | | 1 | - |
| tRNA(Met) | 3845 | 3914 | 70 | | | | 0 | + |
| ND2 | 3915 | 4956 | 1042 | 347 | ATA | T-- | 0 | + |
| tRNA(Trp) | 4957 | 5024 | 68 | | | | 13 | + |
| tRNA(Ala) | 5038 | 5106 | 69 | | | | 1 | - |
| tRNA(Asn) | 5108 | 5179 | 72 | | | | 33 | - |
| tRNA(Cys) | 5213 | 5280 | 68 | | | | 0 | - |
| tRNA(Tyr) | 5281 | 5348 | 68 | | | | 1 | - |
| COX1 | 5350 | 6894 | 1545 | 514 | ATG | TAA | -3 | + |
| tRNA(Ser) | 6892 | 6962 | 71 | | | | 4 | - |

| Gene | Position | | Size | | Codon | | Intergenic Space | |
|---|---|---|---|---|---|---|---|---|
| | Start | End | Nucleotide | Aminoacid | Start | Stop | (bp) | Strand |
| tRNA(Asp) | 6967 | 7034 | 68 | | | | 0 | + |
| COX2 | 7035 | 7718 | 684 | 227 | ATG | TAA | 17 | + |
| tRNA(Lys) | 7736 | 7802 | 67 | | | | 1 | + |
| ATP8 | 7804 | 8007 | 204 | 67 | ATG | TAA | -43 | + |
| ATP6 | 7965 | 8644 | 680 | 226 | ATG | TA- | 0 | + |
| COX3 | 8645 | 9428 | 784 | 261 | ATG | T-- | 0 | + |
| tRNA(Gly) | 9429 | 9496 | 68 | | | | 0 | + |
| ND3 | 9497 | 9842 | 346 | 115 | ATA | T-- | 0 | + |
| tRNA(Arg) | 9843 | 9913 | 71 | | | | 0 | + |
| ND4L | 9914 | 10210 | 297 | 98 | ATG | TAA | -7 | + |
| ND4 | 10204 | 11581 | 1378 | 459 | ATG | T-- | 0 | + |
| tRNA(His) | 11582 | 11650 | 69 | | | | 0 | + |
| tRNA(Ser) | 11651 | 11710 | 60 | | | | 0 | + |
| tRNA(Leu) | 11711 | 11780 | 70 | | | | 0 | + |
| ND5 | 11781 | 13601 | 1821 | 606 | ATA | TAA | -17 | + |
| ND6 | 13585 | 14112 | 528 | 175 | ATG | TAA | 0 | - |
| tRNA(Glu) | 14113 | 14181 | 69 | | | | 4 | - |
| CYTB | 14186 | 15325 | 1140 | 379 | ATG | AGA | 0 | + |
| tRNA(Thr) | 15326 | 15395 | 70 | | | | -3 | + |
| tRNA(Pro) | 15395 | 15460 | 66 | | | | 0 | - |

| Gene | Position | | Size | | Codon | | Intergenic Space | |
|---|---|---|---|---|---|---|---|---|
| | Start | End | Nucleotide | Aminoacid | Start | Stop | (bp) | Strand |
| Control Region | 15461 | 16730 | 1270 | | | | | |
