# Supplementary material for: Mitogenomic sequencing of the Brazilian Mastiff and Brazilian Terrier suggests a complex scenario of breed formation for two established Brazilian dog breeds
Source: Genet Mol Biol. 2026 Apr 17;49(1):e20250149. doi: 10.1590/1678-4685-GMB-2025-0149 (PMC13123249; doi:10.1590/1678-4685-GMB-2025-0149)
Supplement: Table S2 - [file 1415-4757-GMB-49-1-e20250149-s2.pdf]

Genetics and Molecular Biology
https://doi.org/10.1590/1678-4685-GMB-2025-0149

Supplementary Material to "Mitogenomic sequencing of the Brazilian Mastiff and Brazilian Terrier suggests a complex scenario of breed formation for two established Brazilian dog breeds"

Table S2 - Characteristics of the mitochondrial genome of a Brazilian Mastiff dog (*Canis lupus familiaris*).

Gene	Position		Size		Codon		Intergenic Space	
	Start	End	Nucleotides	Aminoacids	Start	Stop	(bp)	Strand
tRNA(Phe)	1	69	69				0	+
12S rRNA	70	1023	954				0	+
tRNA(Val)	1024	1090	67				0	+
16S rRNA	1091	2671	1581				0	+
tRNA(Leu)	2672	2746	75				2	+
ND1	2749	3704	955	317	ATG	TA-	0	+
tRNA(Ile)	3705	3773	69				-4	+
tRNA(Gln)	3770	3844	75				1	-
tRNA(Met)	3846	3915	70				0	+
ND2	3916	4957	1042	346	ATA	T--	0	+
tRNA(Trp)	4958	5025	68				13	+
tRNA(Ala)	5039	5107	69				1	-
tRNA(Asn)	5109	5180	72				33	-
tRNA(Cys)	5214	5281	68				0	-
tRNA(Tyr)	5282	5349	68				1	-
COX1	5351	6895	1545	514	ATG	TAA	-3	+
tRNA(Ser)	6893	6963	71				4	-
tRNA(Asp)	6968	7035	68				0	+
COX2	7036	7719	684	227	ATG	TAA	17	+
tRNA(Lys)	7737	7803	67				1	+

Gene	Position		Size		Codon		Intergenic Space	
	Start	End	Nucleotides	Aminoacids	Start	Stop	(bp)	Strand
ATP8	7805	8008	204	67	ATG	TAA	-43	+
ATP6	7966	8645	680	226	ATG	TA-	0	+
COX3	8646	9429	784	261	ATG	T--	0	+
tRNA(Gly)	9430	9497	68				0	+
ND3	9498	9843	346	115	ATA	T--	0	+
tRNA(Arg)	9844	9914	71				0	+
ND4L	9915	10211	297	98	ATG	TAA	-7	+
ND4	10205	11582	1378	459	ATG	T--	0	+
tRNA(His)	11583	11651	69				0	+
tRNA(Ser)	11652	11711	60				0	+
tRNA(Leu)	11712	11781	70				0	+
ND5	11782	13602	1821	606	ATA	TAA	-17	+
ND6	13586	14113	528	175	ATG	TAA	0	-
tRNA(Glu)	14114	14182	69				4	-
CYTB	14187	15326	1140	379	ATG	AGA	0	+
tRNA(Thr)	15327	15396	70				-1	+
tRNA(Pro)	15396	15461	66				0	-
Control Region	15462	16732	1271					
