# Supplementary material for: Mitogenomic sequencing of the Brazilian Mastiff and Brazilian Terrier suggests a complex scenario of breed formation for two established Brazilian dog breeds
Source: Genet Mol Biol. 2026 Apr 17;49(1):e20250149. doi: 10.1590/1678-4685-GMB-2025-0149 (PMC13123249; doi:10.1590/1678-4685-GMB-2025-0149)
Supplement: Table S3 - [file 1415-4757-GMB-49-1-e20250149-s3.pdf]

Genetics and Molecular Biology
https://doi.org/10.1590/1678-4685-GMB-2025-0149

Supplementary Material to "Mitogenomic sequencing of the Brazilian Mastiff and Brazilian Terrier suggests a complex scenario of breed formation for two established Brazilian dog breeds"

Table S3 - Diagnostic mutations supporting mitochondrial haplogroup assignments.

Breed	Accession #	Diagnosed Haplogroup	Diagnostic SNP	SNP Haplogroup
B. Terrier	MH105046	A1a1b2	8536	A1a1b
			8807A	A1a1b
			10165	A1a1b
			**14474**	**A1a1b2**
B. Mastiff	MH105047	B1a1a	16	BCE
			381A	BCE
			445G	B1
			1756	BCE
			2185	B
			2812	BE
			3028C	B1a
			4204A	B
			4277G	BE
			4390	BE
			4646	B1
			6764	B
			7014	B1a
			8101A	B1a
			8221C	BCE
			8569G	BE
			8736	BE
			8760G	BCE

Breed	Accession #	Diagnosed Haplogroup	Diagnostic SNP	SNP Haplogroup
			8817G	BE
			8877G	BCE
			9219G	B1
			9252	B1a1
			9825A	B1
			10257A	B1
			10440	BE
			10542G	B1
			11176	B1a
			11948G	B
			**12459A**	**B1a1a**
			12665	BE
			13594A	B
			13618G	BCE
			14671A	BE
			14930	B1
			15612	B
			15632	BE
			15639G	B
			15643G	B
			15652A	BE
			15815	B
			15955	BCE
			16003G	BCE
			16672	B1

* Diagnostic mutations supporting mitochondrial haplogroup assignments for each Brazilian breed. Fregel *et al.* (2015) was followed for the diagnostic SNPs and the mutation nomenclature was based on the adaptation from Pereira *et al.* (2004) by Fregel *et al.* (2015). B. Terrier shows less SNPs because it is part of the same A1a1 haplogroup as the reference genome, so, when comparing the two, only the SNPs that differentiate the reference's A1a1a from the B. Terrier's A1a1b* groups appear. Bold SNP position and diagnostic represent the most specific assignment.
