# Supplementary material for: Mitogenomic sequencing of the Brazilian Mastiff and Brazilian Terrier suggests a complex scenario of breed formation for two established Brazilian dog breeds
Source: Genet Mol Biol. 2026 Apr 17;49(1):e20250149. doi: 10.1590/1678-4685-GMB-2025-0149 (PMC13123249; doi:10.1590/1678-4685-GMB-2025-0149)
Supplement: Table S4 - [file 1415-4757-GMB-49-1-e20250149-s4.pdf]

Genetics and Molecular Biology
https://doi.org/10.1590/1678-4685-GMB-2025-0149

Supplementary Material to "Mitogenomic sequencing of the Brazilian Mastiff and Brazilian Terrier suggests a complex scenario of breed formation for two established Brazilian dog breeds"

Table S4 - Comparison between SNPs of the Melanophilin gene of a Brazilian Terrier dog and other breeds (*Canis lupus familiaris*).

**Position**	**Doberman Pinscher (European)/ Large Munsterlander/Beagle**	**Doberman Pinscher (North American)**	**German Pinscher**	**Brazilian Terrier**	**Original (*Canis lupus*)**
**157354 (exon 1)**	G	G	G	n.d.	G
**157471 (exon 1)**	A	A	A	n.d.	A
157486 (intron 1)	A	A	A	n.d.	A
163882 (intron 1)	C	C	T	C	C
163889 (intron 1)	A	A	C	A	A
163936 (intron 1)	G	G	A	G	G
163983 (intron 1)	G	G	A	G	G
164012 (intron 1)	G	G	A	A	G
164049 (intron 1)	G	G	A	G	G
164075 (intron 1)	T	T	C	T	T
**164258 (exon 2)**	T	T	C	n.d.	T
164397 (intron 2)	G	G	A	G	G
**180908 (exon 3)**	G	G	A	n.d.	G
**180994 (exon 3)**	C	T	C	C	T
**180998 (exon 3)**	G	G	A	A	G
183047 (intron 4)	C	G	G	G	C
**183254 (exon 5)**	C	T	C	C	G
183336 (intron 5)	C	T	C	n.d.	C
184650 (intron 5)	C	T	C	Y	C
**184751 (exon 6)**	G	G	G	n.d.	G
184818 (intron 6)	G	A	G	G	A

Position	Doberman Pinscher (European)/ Large Munsterlander/Beagle	Doberman Pinscher (North American)	German Pinscher	Brazilian Terrier	Original (*Canis lupus*)
186072 (intron 6)	T	C	T	n.d.	T
186091 (intron 6)	T	C	T	n.d.	T
**186184 (exon 7)**	A	G	A	n.d.	A
186329 (intron 7)	G	A	G	n.d.	G
**187449..54 (exon 8)**	GAGGAG	del	GAGGAG	GAGGAG	GAGGAG
189560 (intron 8)	G	G	G	G	G
189575 (intron 8)	C	A	C	C	A
**189719 (exon 9)**	C	C	C	n.d.	C
**189728 (exon 9)**	n.d.	G	A	n.d.	n.d.
195548 (intron 9)	n.d.	G	G	n.d.	G
**195720 (exon 10)**	n.d.	G	A	A	G
**195808 (exon 10)**	n.d.	A	G	G	A
195888 (intron 10)	n.d.	G	G	n.d.	G
195891 (intron 10)	n.d.	G	G	n.d.	G
195900 (intron 10)	n.d.	G	G	n.d.	G
195901 (intron 10)	n.d.	G	del	n.d.	T
195932 (intron 10)	n.d.	G	G	n.d.	G
**196097 (exon 11)**	n.d.	G	G	n.d.	G
196354 (intron 11)	n.d.	T	C	C	T
197343 (intron 11)	n.d.	T	T	T	T
197510 (intron 11)	n.d.	C	C	n.d.	C
197673 (intron 12)	n.d.	C	C	C	C
197730 (intron 12)	n.d.	G	G	G	G
198080 (intron 12)	n.d.	A	n.d.	G	A
198126 (intron 12)	n.d.	A	n.d.	G	A
199847 (intron 13)	n.d.	A	A	R (A or G)	A
**202837 (exon 16)**	n.d.	T	C	n.d.	C
